# Real-world safety and effectiveness of nivolumab in patients with cancer of unknown primary: results from a nationwide post-marketing surveillance in Japan

**DOI:** 10.1007/s10147-026-03147-3

**Published:** 2026-08-26

**Authors:** Akira Kawasaki, Akito Kakuuchi, Ayumi Akamatsu, Misato Nakanishi, Chikara Honda

**Affiliations:** 1 Pharmacovigilance Division, Ono Pharmaceutical Co., Ltd., 1-8-2 Kyutaromachi, Chuo-ku, Osaka, 541-8564 Japan; 2 Oncology Medical Affairs, Ono Pharmaceutical Co., Ltd., Osaka, Japan

**Keywords:** Cancer of unknown primary, Immunotherapy, Nivolumab, Post-marketing surveillance, Real-world evidence

## Abstract

**Background:**

Cancer of unknown primary (CUP) is associated with poor prognosis. In December 2021, nivolumab was approved in Japan for the treatment of CUP based on a small phase II trial. This post-marketing surveillance (PMS) evaluated the real-world safety and effectiveness of nivolumab in Japanese patients with CUP.

**Methods:**

This prospective, observational, PMS enrolled patients with CUP who initiated nivolumab between April 2022 and June 2023 at 66 institutions. Patients were observed for 6 months. Safety outcomes were treatment-related adverse events (TRAEs), and effectiveness outcomes included overall response rate (ORR) and overall survival (OS). Analyses were descriptive.

**Results:**

Among 151 patients in the safety set, the median age was 70 years and 79.5% had an Eastern Cooperative Oncology Group performance status (PS) 0–1. Any grade and Grade ≥ 3 TRAEs occurred in 25.2% and 11.9% of patients, respectively. Frequently reported TRAEs included interstitial lung disease (3.3%), hepatic function abnormal (2.6%), and hypothyroidism (2.6%). There was one TRAE (neutropenia) resulting in death. In the effectiveness set (*n* = 144), the ORR was 21.5%. ORR was higher in patients without prior systemic therapy (25.4% vs. 18.2%) and in those with lymph node-only involvement (36.6% vs. 15.5%). The 6-month OS rate was 71.0%, and median OS was not reached. The 6-month OS rates were greater in patients with PS 0–1 and lymph node-only involvement.

**Conclusions:**

Nivolumab was tolerated in patients with CUP in routine practice in Japan, with no new safety concerns, and indicated antitumor activity consistent with clinical trial results.

**Trial registration:**

Japan Registry of Clinical Trials (jRCT2041240121).

**Supplementary Information:**

The online version contains supplementary material available at https://doi.org/10.1007/s10147-026-03147-3.

## Introduction

Cancer of unknown primary (CUP) is defined as a malignant tumor in which the primary lesion cannot be identified despite thorough diagnostic evaluation and is histologically confirmed to be metastatic [1]. CUP accounts for 2–5% of all carcinomas, making it a substantial clinical problem [2–4].

CUP is classified into good- and poor-prognosis groups, which differ in treatment methods and clinical outcomes. 80–85% of patients with CUP belong to the poor-prognosis group [2]. The median overall survival (OS) of the poor-prognosis group ranges from several months to 1 year [1, 2]. This poor prognosis has remained unchanged for decades, underscoring the need for new therapeutic options [2].

Immune checkpoint inhibitors (ICIs) have transformed the treatment landscape across multiple malignancies. Nivolumab, a humanized IgG4 monoclonal antibody that targets programmed cell death protein 1 (PD-1), has emerged as a cornerstone of modern immunotherapy. Nivolumab has been approved in Japan for a wide range of malignancies, including malignant melanoma, non-small cell lung cancer (NSCLC), classic Hodgkin lymphoma, head and neck cancer, gastric cancer, malignant pleural mesothelioma, other malignant mesotheliomas, microsatellite instability-high colorectal cancer, esophageal cancer, urothelial carcinoma, cutaneous epithelial malignancies, and hepatocellular carcinoma [5, 6].

Based on the biological hypothesis of CUP development and on findings from immune-related gene expression profiling [4, 7, 8], a landmark clinical study of nivolumab for CUP was conducted (NM-K2002 study) [9], which led to its approval in December 2021 for the treatment of CUP in Japan. However, the study was limited to 56 patients with an Eastern Cooperative Oncology Group (ECOG) performance status (PS) of 0–1 and required an estimated survival of  ≥ 90 days prior to starting nivolumab [9]. Therefore, the extrapolation of its findings to real-world CUP populations remains uncertain.

To address this, a post-marketing surveillance (PMS) was conducted to evaluate these aspects in patients with CUP.

## Methods

### Study design and setting

This prospective, non-interventional, observational, PMS evaluated the safety and effectiveness of nivolumab in patients with CUP in Japan. Patients were observed for 6 months after their first nivolumab administration, regardless of treatment continuation. Nivolumab was administered intravenously at a dose of 240 mg every 2 weeks or 480 mg every 4 weeks.

The study was conducted in accordance with Japanese Good Post-Marketing Study Practice regulations. Approval from the institutional review board or ethics committee was obtained. The PMS was registered in the Japan Registry of Clinical Trials (jRCT2041240121).

### Participants

Case report forms (CRFs) were collected for all patients with CUP for whom nivolumab was newly initiated between April 18, 2022, and June 30, 2023, at 66 participating institutions that permitted the publication of data. In accordance with the product label, however, nivolumab is contraindicated in patients with a history of serious hypersensitivity to the drug or its components and in pregnant women [6]. All eligible patients were enrolled consecutively and registered centrally.

### Data collection and endpoints

Participating physicians recorded information including baseline variables (age, sex, ECOG PS, medical history, CUP histologic type, lesion sites, and prior treatments for CUP), nivolumab administration status (dose, number of administrations, reasons for discontinuation), safety, and clinical outcomes. For the categorization of lesion sites, “lymph node-only” was defined as the presence of lesions exclusively in the lymph nodes. “Other sites” denoted the presence of lesions in any non-lymph node location, with or without concurrent lymph node involvement.

The primary outcome was the occurrence of treatment-related adverse events (TRAEs), including TRAEs of special interest, as classified using the Medical Dictionary for Regulatory Activities (MedDRA) Japanese version 27.1 and graded by the National Cancer Institute Common Terminology Criteria for Adverse Events (CTCAE) version 5.0 (Japanese Clinical Oncology Group version). TRAEs were evaluated in all patients and in subgroups stratified by baseline characteristics. Each TRAE of special interest comprised an aggregation of TRAEs with related preferred terms.

Effectiveness outcomes included the best overall response within 6 months, assessed by the treating physician based on Response Evaluation Criteria in Solid Tumors (RECIST) version 1.1 (complete response [CR], partial response [PR], stable disease [SD], progressive disease [PD], and not evaluable [NE]); overall response rate (ORR; CR + PR); disease control rate (DCR; CR + PR + SD); and overall survival (OS) from Day 1 to death or censoring (at 183 days).

### Statistical methods

A registration target of 120 patients was set based on results of the NM-K2002 study [9] and accounting for potential dropouts.

The safety analysis set (SAF) comprised all enrolled patients who met eligibility criteria, received at least one dose of nivolumab, and for whom safety data were available. The effectiveness analysis set (EAS) comprised patients in the SAF who met additional criteria, including a planned tumor response evaluation and no major deviations from approved nivolumab dosing, and who did not receive concomitant antineoplastic agents during the observation period.

Analyses were descriptive. Categorical variables were summarized as frequencies and percentages. Continuous variables were summarized using descriptive statistics (mean, standard deviation, median, minimum, and maximum). For ORR and DCR, the proportions were calculated for the overall EAS and stratified by baseline characteristics. OS was estimated using the Kaplan–Meier method, with censoring at the last known alive date or Day 183, whichever occurred first. Missing or unknown data were analyzed without imputation. All analyses were performed using SAS software version 9.4 (SAS Institute Inc., Cary, NC, USA).

## Results

### Patient disposition

**Fig. 1:**
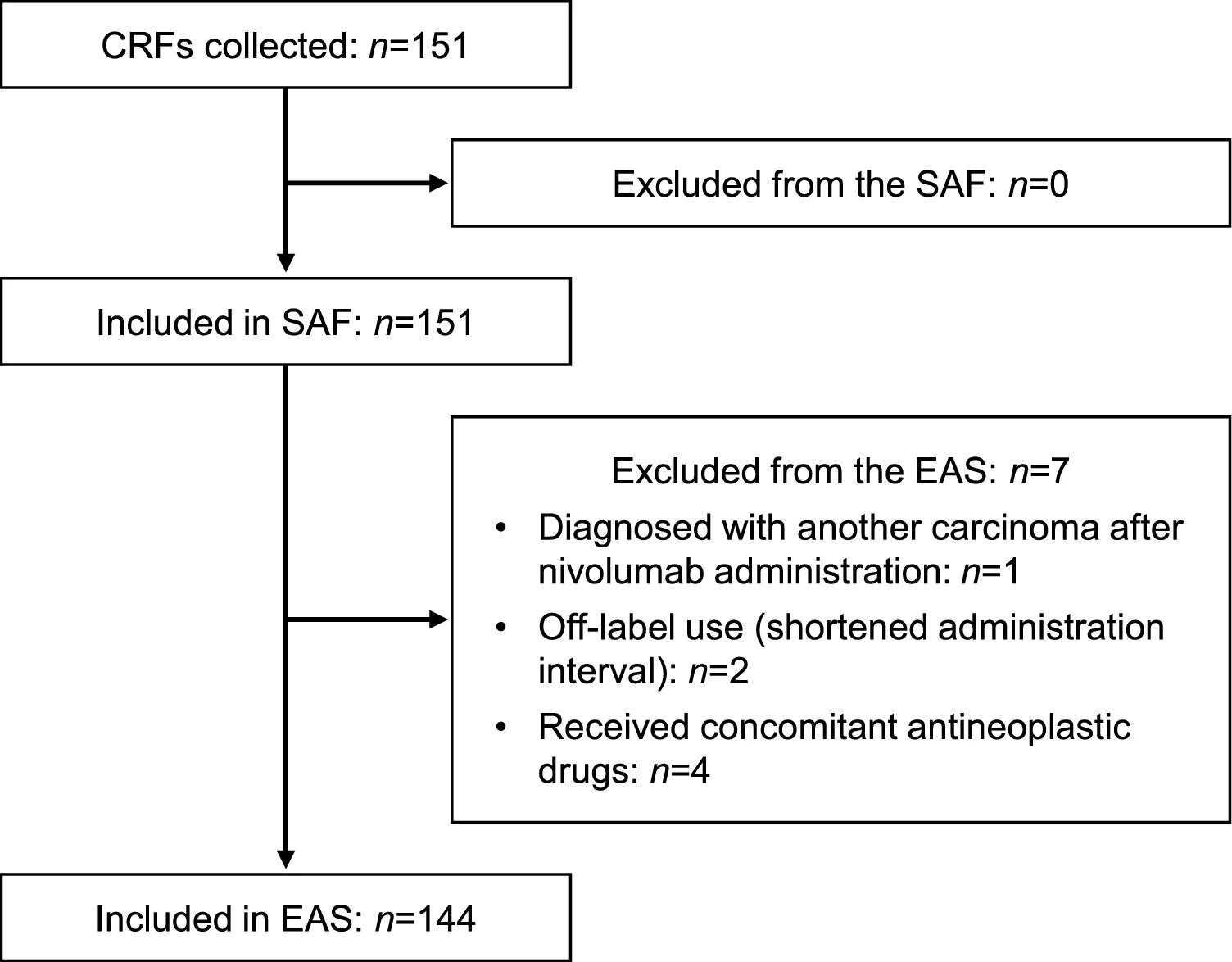
Patient disposition. *CRF* case report form, *EAS* effectiveness analysis set, *SAF* safety analysis set

Between April 18, 2022, and June 30, 2023, a total of 151 patients with CUP were registered from 66 medical institutions (Fig. 1). All registered patients met the eligibility criteria and were included in the SAF. Seven patients were excluded from the EAS: one was diagnosed with another carcinoma after nivolumab administration, two received off-label short-term nivolumab treatment, and four received concomitant antineoplastic drugs. The EAS, therefore, comprised 144 patients.

### Baseline characteristics

In the SAF (*n* = 151), the median age was 70 years (range, 26–89 years) and 54 patients (35.8%) were aged ≥ 75 years (Table 1). ECOG PS was 0–1 in 120 patients (79.5%). The most common histologic type was poorly differentiated adenocarcinoma/undifferentiated carcinoma (*n* = 47; 31.1%), followed by well/moderately differentiated adenocarcinoma (*n* = 39; 25.8%), squamous cell carcinoma (*n* = 23; 15.2%), poorly differentiated malignant neoplasm (*n* = 18; 11.9%), neuroendocrine tumor (*n* = 2; 1.3%), and other histologic types (*n* = 22; 14.6%). At the start of nivolumab treatment, 41 patients (27.2%) had lesions confined to lymph nodes only. Seventy-three patients (48.3%) had not received any prior systemic therapy.

**Table 1:** Baseline characteristics (*n* = 151)

Characteristic		Patients (%)
Sex	Male	86 (57.0)
	Female	65 (43.0)
ECOG PS	0–1	120 (79.5)
	2	26 (17.2)
	3–4	5 (3.3)
Age, years	< 75 years	97 (64.2)
	≥ 75 years	54 (35.8)
	Mean (SD)	68.1 (11.9)
	Median (range)	70 (26–89)
Medical history^a^	Lung disease	7 (4.6)
	Liver disease	10 (6.6)
	Kidney disease	10 (6.6)
	Thyroid disease	6 (4.0)
	Autoimmune disease	13 (8.6)
	Other diseases	68 (45.0)
Tumor histologic type	Well/moderately differentiated adenocarcinoma	39 (25.8)
	Poorly differentiated adenocarcinoma/undifferentiated carcinoma	47 (31.1)
	Squamous cell carcinoma	23 (15.2)
	Neuroendocrine tumor	2 (1.3)
	Poorly differentiated malignant neoplasm	18 (11.9)
	Other tumors	22 (14.6)
Lesion site at the start of nivolumab	Lymph node-only involvement^b^	41 (27.2)
	Other sites^c^	110 (72.8)
Lesion site at the start of nivolumab by organ^d^	Lymph node	85 (56.3)
	Mediastinum	7 (4.6)
	Lung	16 (10.6)
	Pleura	13 (8.6)
	Peritoneum	53 (35.1)
	Retroperitoneum	4 (2.6)
	Liver	23 (15.2)
	Bone	32 (21.2)
	Brain	2 (1.3)
	Other	24 (15.9)
Prior systemic therapy before nivolumab	No	73 (48.3)
	Yes	78 (51.7)

Values are *n* (%), unless specified otherwise

^a^The number of patients for whom medical history was available was 150 for all categories, except “other”, for which data were available for all 151 patients

^b^Patients with no lesions at sites other than lymph node(s)

^c^“Other sites” denotes lesions at sites other than lymph nodes, with or without lymph node involvement

^d^If a patient had multiple lesion sites, the patient was counted for each site

*ECOG PS* Eastern Cooperative Oncology Group performance status, *SD* standard deviation

### Nivolumab exposure

The median number of nivolumab administrations was 5.0 (range 1–14) (Supplementary Table S1). The median treatment duration was 73.0 days (range 1–183). At 6 months, 40 patients (26.5%) were still receiving nivolumab, while 111 patients (73.5%) had discontinued treatment. The most common reason for discontinuation was disease progression, including death, in 64 patients (57.7%). Other reasons included lack of expected efficacy (26.1%), occurrence of AEs (19.8%), transfer to another facility (6.3%), and other reasons (5.4%).

### Safety

**Fig. 2:**
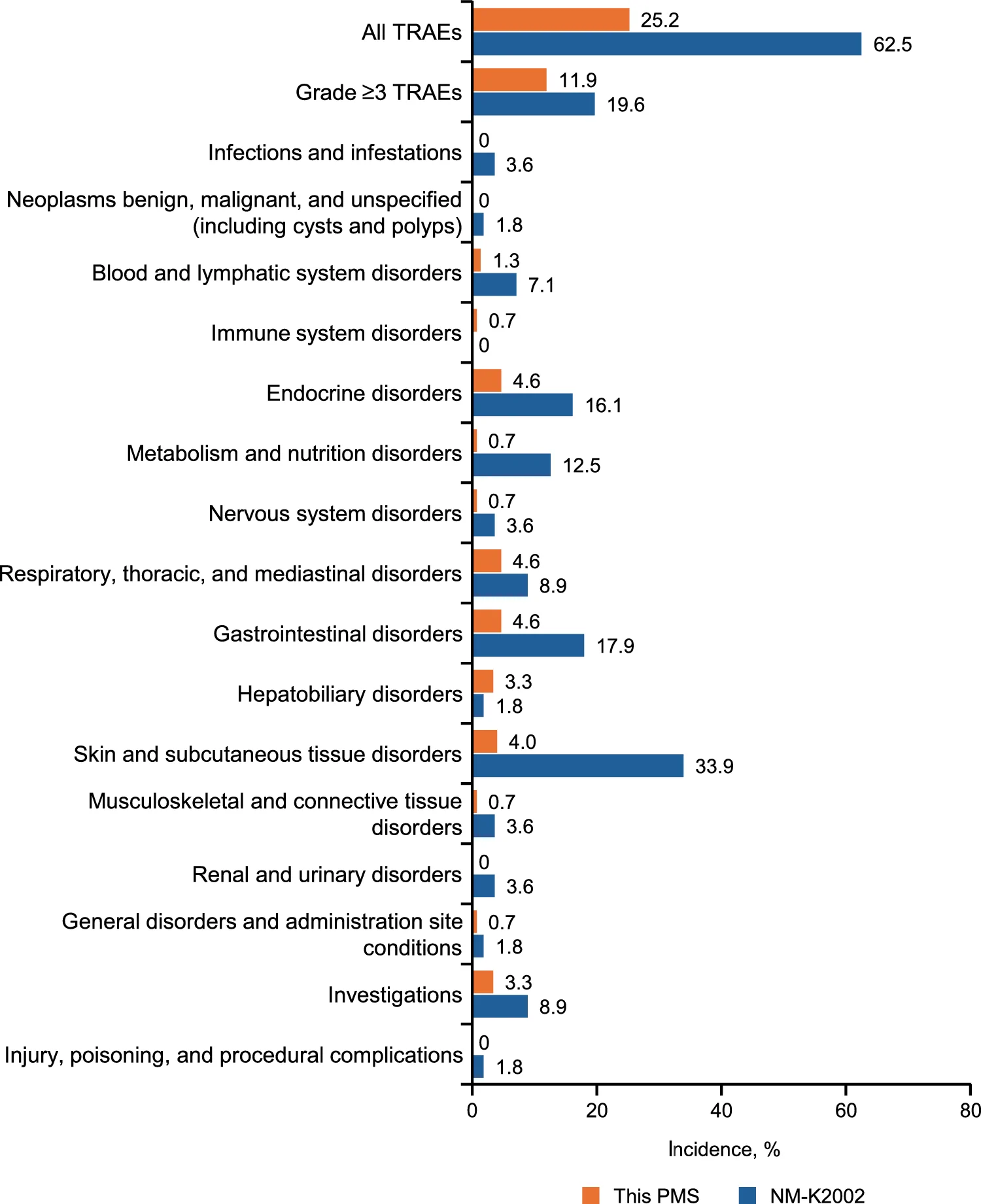
Comparison of TRAE incidence between this PMS and NM-K2002. *PMS* post-marketing surveillance, *TRAE* treatment-related adverse event

In the SAF, TRAEs of any grade occurred in 38 patients (25.2%) and Grade ≥ 3 TRAEs were reported in 18 patients (11.9%) (Table 2). At the System Organ Class level, the incidences of hepatobiliary disorders and immune system disorders were numerically higher in this PMS (3.3% and 0.7%, respectively) than in the NM-K2002 study (1.8% and 0%, respectively; Fig. 2). At the Preferred Term, events occurring in ≥ 2.5% of patients included interstitial lung disease (ILD) (3.3%), hepatic function abnormal (2.6%), and hypothyroidism (2.6%). The most common Grade ≥ 3 TRAEs were hepatic function abnormal (2.0%), ILD (1.3%), and stomatitis (1.3%). One treatment-related death occurred due to neutropenia.

**Table 2:** TRAEs according to SOC and PT (*n* = 151)

SOC and PT	Any grade	Grade ≥ 3
All	38 (25.2)	18 (11.9)
Blood and lymphatic system disorders	2 (1.3)	2 (1.3)
Eosinophilia	1 (0.7)	1 (0.7)
Neutropenia	1 (0.7)	1 (0.7)
Immune system disorders	1 (0.7)	1 (0.7)
Cytokine release syndrome	1 (0.7)	1 (0.7)
Endocrine disorders	7 (4.6)	2 (1.3)
Adrenal insufficiency	2 (1.3)	1 (0.7)
Hyperthyroidism	1 (0.7)	0
Hypothyroidism	4 (2.6)	0
Secondary adrenocortical insufficiency	1 (0.7)	1 (0.7)
Metabolism and nutrition disorders	1 (0.7)	1 (0.7)
Acidosis	1 (0.7)	1 (0.7)
Type 1 diabetes mellitus	1 (0.7)	1 (0.7)
Nervous system disorders	1 (0.7)	1 (0.7)
Encephalitis autoimmune	1 (0.7)	1 (0.7)
Respiratory, thoracic and mediastinal disorders	7 (4.6)	2 (1.3)
ILD	5 (3.3)	2 (1.3)
Pneumonitis	2 (1.3)	0
Gastrointestinal disorders	7 (4.6)	3 (2.0)
Colitis	1 (0.7)	0
Diarrhea	1 (0.7)	0
Nausea	1 (0.7)	0
Stomatitis	3 (2.0)	2 (1.3)
Immune-mediated enterocolitis	1 (0.7)	1 (0.7)
Hepatobiliary disorders	5 (3.3)	3 (2.0)
Cholangitis	1 (0.7)	0
Hepatic function abnormal	4 (2.6)	3 (2.0)
Skin and subcutaneous tissue disorders	6 (4.0)	0
Dry skin	1 (0.7)	0
Eczema nummular	1 (0.7)	0
Pruritus	1 (0.7)	0
Rash	2 (1.3)	0
Immune-mediated dermatitis	1 (0.7)	0
Musculoskeletal and connective tissue disorders	1 (0.7)	1 (0.7)
Rheumatic disorder	1 (0.7)	1 (0.7)
General disorders and administration site conditions	1 (0.7)	0
Fatigue	1 (0.7)	0
Investigations	5 (3.3)	2 (1.3)
Alanine aminotransferase increased	1 (0.7)	1 (0.7)
Amylase increased	1 (0.7)	0
Aspartate aminotransferase increased	3 (2.0)	1 (0.7)
Blood creatinine increased	2 (1.3)	1 (0.7)

Values are *n* (%)

*ILD* interstitial lung disease, *PT* Preferred Term, *SOC* System Organ Class, *TRAE* treatment-related adverse event

In subgroup analyses, the incidences of TRAEs were 25.0% in patients with an ECOG PS of 0–1 and 25.8% in those with an ECOG PS of 2–4, with rates of Grade ≥ 3 TRAEs of 12.5% and 9.7%, respectively. By age, the incidences of TRAEs were 33.3% in patients aged ≥ 75 years and 20.6% in those aged < 75 years, with Grade ≥ 3 TRAEs occurring in 18.5% and 8.2%, respectively. Among patients aged ≥ 75 years, ILD (7.4%), hepatic function abnormal (3.7%), and hypothyroidism (3.7%) were the most frequently observed TRAEs. The incidences of TRAEs in patients with a history of pulmonary disease were 71.4% for any grade TRAEs and 57.1% for Grade  ≥ 3 TRAEs, compared with 23.1% and 9.8% in those without such a history. In patients with a history of thyroid disease, the incidences were 66.7% for any grade TRAEs and 16.7% for Grade ≥ 3 TRAEs, compared with 23.6% and 11.8% in those without such a history (Supplementary Table S2).

TRAEs of special interest with an incidence of  ≥ 3% included hepatitis-related events (hepatitis fulminant, hepatic failure, hepatic function disorder, hepatitis, and cholangitis sclerosing), which occurred in 5.3% of patients (Grade ≥ 3 TRAEs: 2.6%), ILD in 4.6% (Grade ≥ 3 TRAEs: 1.3%), and endocrine disorders (e.g., thyroid dysfunction, pituitary insufficiency, and adrenal disorder) in 4.6% (Grade ≥ 3 TRAEs: 1.3%) (Table 3). Recovery or improvement was reported in 75.0% of patients with hepatitis-related TRAEs, all patients with ILD, and in 28.6% of patients with endocrine disorders (Supplementary Table S3). In addition, encephalitis and meningitis (0.7%), and type 1 diabetes mellitus (0.7%) were observed in patients with CUP in this PMS. For each of these categories, Grade  ≥ 3 TRAEs occurred in 0.7% of patients. The patient with encephalitis and meningitis recovered with sequelae, and the patient with type 1 diabetes mellitus had not recovered.

**Table 3:** TRAEs listed in the safety specification and new safety findings (*n* = 151)

	Any grade	Grade ≥ 3
ILD^a^	7 (4.6)	2 (1.3)
Myasthenia gravis, myocarditis, myositis, and rhabdomyolysis	0	0
Colitis, enteritis, and severe diarrhea^b^	2 (1.3)	0
Type 1 diabetes mellitus	1 (0.7)	1 (0.7)
Hepatic failure, hepatic disorder, hepatitis, cholangitis sclerosing^c^	8 (5.3)	4 (2.6)
Endocrine disorders (thyroid dysfunction, pituitary insufficiency, and adrenal disorder)^d^	7 (4.6)	2 (1.3)
Neuropathy	0	0
Renal disorder	2 (1.3)	1 (0.7)
Encephalitis and meningitis	1 (0.7)	1 (0.7)
Severe skin disorder	0	0
Venous thromboembolism	0	0
Infusion reaction^e^	4 (2.6)	1 (0.7)
Serious blood disorder	0	0
Hemophagocytic syndrome	0	0
Tuberculosis	0	0
Pancreatitis	0	0
Severe gastritis	0	0
Uveitis	0	0
Cardiac disorders such as atrial fibrillation, bradycardia, and ventricular extrasystoles	0	0
Aplasia pure red cell	0	0
Tumor hemorrhage	0	0
Fistula	0	0

Values are *n* (%)

^a^Comprises the preferred terms “interstitial lung disease” and “pneumonitis”

^b^Comprises the preferred terms “colitis” and “diarrhea”

^c^Comprises the preferred terms “alanine aminotransferase increased”, “aspartate aminotransferase increased”, “cholangitis”, and “hepatic function abnormal”

^d^Comprises the preferred terms “adrenal insufficiency”, “hyperthyroidism”, “hypothyroidism”, and “secondary adrenal insufficiency”

^e^Comprises the preferred terms “pruritus”, “rash”, and “cytokine release syndrome”

*ILD* interstitial lung disease, *TRAE* treatment-related adverse event

### Effectiveness

Among the 144 patients in the EAS, the best overall responses were CR in 1.4%, PR in 20.1%, SD in 25.0%, PD in 45.1%, and NE in 8.3%. The ORR was 21.5% (95% confidence interval [CI], 15.1–29.1) and the DCR was 46.5% (95% CI, 38.2–55.0).

**Fig. 3:**
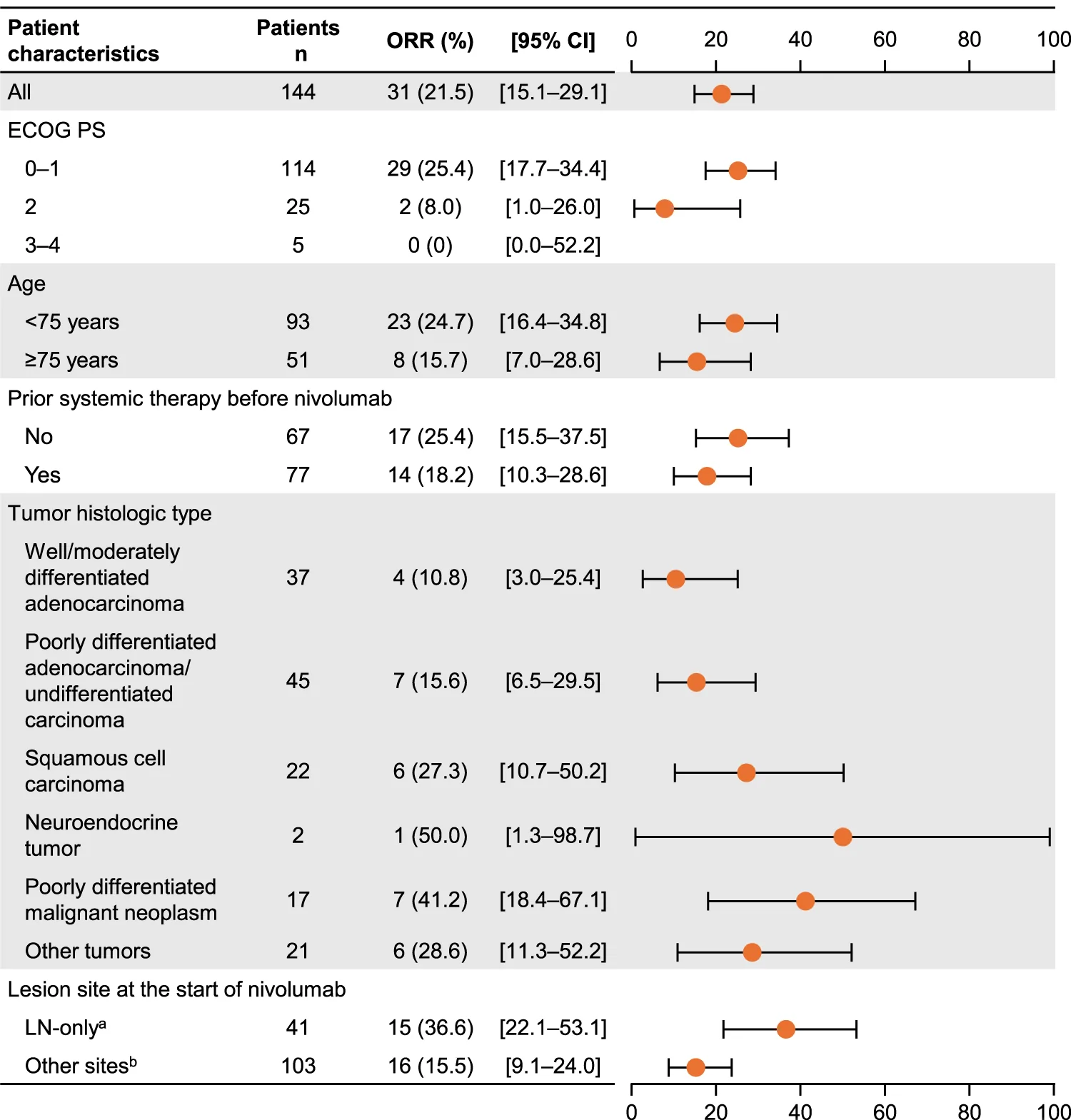
ORR according to baseline patient characteristics in patients with CUP who received nivolumab (*n* = 144). ^a^ “LN-only” denotes lymph node-only involvement, defined as no lesions at sites other than lymph node(s). ^b^ “Other sites” denotes lesions at sites other than lymph nodes, with or without lymph node involvement. *CI* confidence interval, *CUP* cancer of unknown primary, *ECOG PS* Eastern Cooperative Oncology Group performance status, *LN* lymph node, *ORR* overall response rate

By ECOG PS, the ORR was 25.4% (95% CI, 17.7–34.4) for PS 0–1, 8.0% (95% CI, 1.0–26.0) for PS 2, and 0% (95% CI, 0.0–52.2) for PS 3–4. By age group, the ORR was 24.7% (95% CI, 16.4–34.8) in patients aged < 75 years and 15.7% (95% CI, 7.0–28.6) in those aged ≥ 75 years. The ORR was 25.4% (95% CI, 15.5–37.5) without prior systemic therapy and 18.2% (95% CI, 10.3–28.6) with prior systemic therapy. By histology, the ORR was 10.8% (95% CI, 3.0–25.4) for well/moderately differentiated adenocarcinoma, 15.6% (95% CI, 6.5–29.5) for poorly differentiated adenocarcinoma/undifferentiated carcinoma, 27.3% (95% CI, 10.7–50.2) for squamous cell carcinoma, 50.0% (95% CI, 1.3–98.7) for neuroendocrine tumor, 41.2% (95% CI, 18.4–67.1) for poorly differentiated malignant neoplasm, and 28.6% (95% CI, 11.3–52.2) for other tumors. The ORR was 36.6% (95% CI, 22.1–53.1) among patients with lymph node-only involvement compared with 15.5% (95% CI, 9.1–24.0) in patients with disease at other sites, with or without lymph node involvement (Fig. 3). The corresponding DCRs in these subgroups are also presented in Supplementary Table S4.

**Fig. 4:**
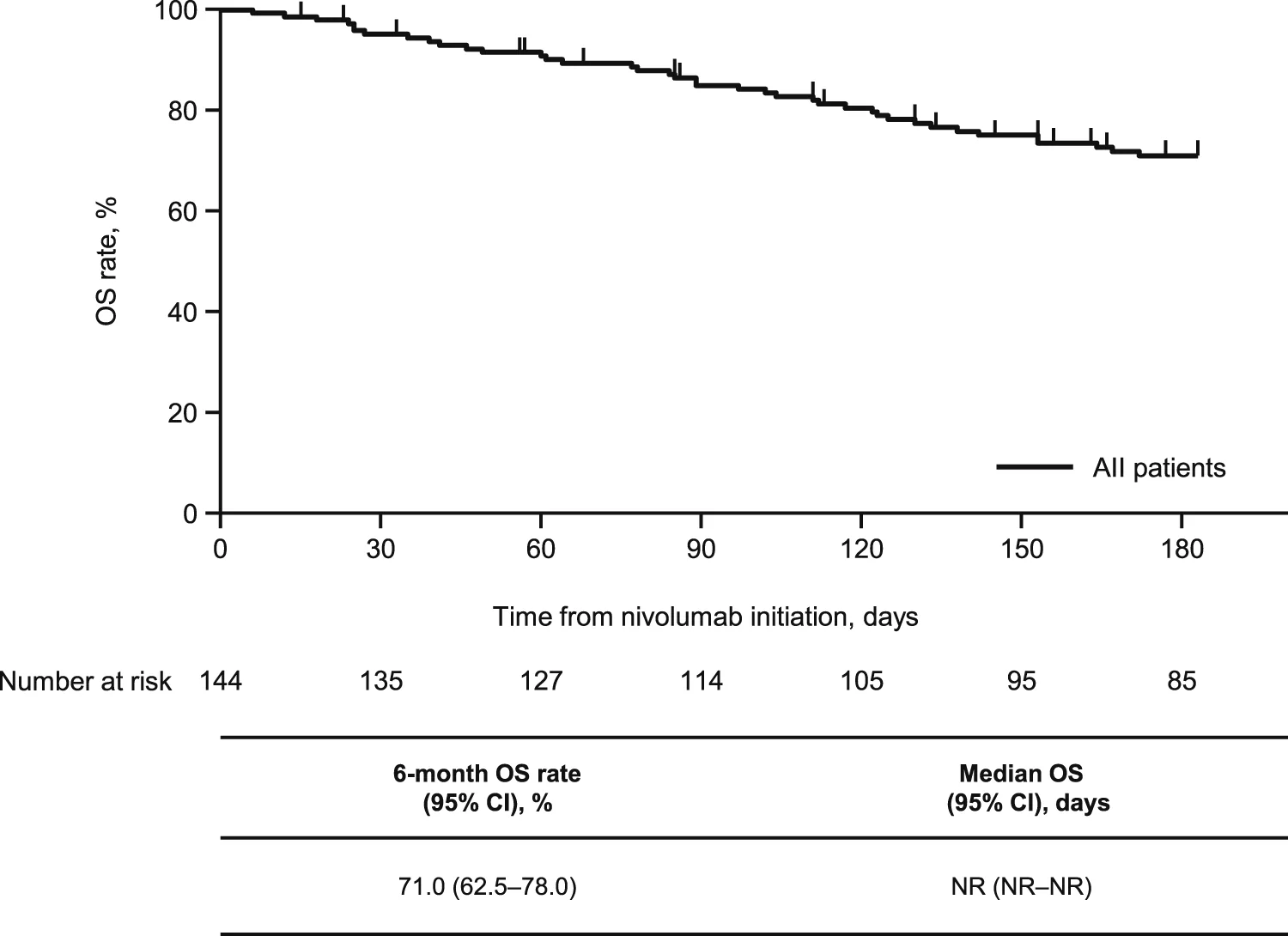
Kaplan–Meier plot of OS in patients with CUP who received nivolumab (*n* = 144). *CI* confidence interval, *CUP* cancer of unknown primary, *NR* not reached, *OS* overall survival

The 6-month OS rate was 71.0% (95% CI, 62.5–78.0) among all patients, and the median OS was not reached (Fig. 4). By ECOG PS, the 6-month OS rate was 81.2% (95% CI, 72.3–87.4) for ECOG PS 0–1 (median not reached), 36.6% (95% CI, 17.0–56.5) for ECOG PS 2 (median 125.0 days [95% CI, 60.0–not reached]), and not reached for ECOG PS 3–4 (median 24.0 days [95% CI, 6.0–not reached]) (Supplementary Figure S1). By age, the 6-month OS rate was 72.6% (95% CI, 61.9–80.7) for patients < 75 years and 68.4% (95% CI, 53.0–79.7) for those aged ≥ 75 years (Supplementary Figure S2). By treatment history, the 6-month OS rate was 72.4% (95% CI, 59.3–81.9) without prior systemic therapy and 69.9% (95% CI, 58.0–79.1) with prior systemic therapy (Supplementary Figure S3). By histology, the 6-month OS rates were 63.2% (95% CI, 45.1–76.8) for well/moderately differentiated adenocarcinoma, 61.9% (95% CI, 44.7–75.2) for poorly differentiated adenocarcinoma/undifferentiated carcinoma, 81.8% (95% CI, 58.5–92.8) for squamous cell carcinoma, 100.0% (95% CI, 100.0–100.0) for neuroendocrine tumor, 81.1% (95% CI, 51.9–93.5) for poorly differentiated malignant neoplasm, and 79.9% (95% CI, 54.8–92.0) for other tumors (Supplementary Figure S4). Patients with lymph node-only involvement had a 6-month OS rate of 92.4% (95% CI, 78.3–97.5) compared with 62.0% (95% CI, 51.3–71.0) in patients with disease at other sites, with or without lymph node involvement (Supplementary Figure S5).

## Discussion

The objective of this PMS was to evaluate the real-world safety of nivolumab in patients with CUP in Japan and its effectiveness was also examined as part of the investigation. This PMS provides the first comprehensive dataset on ICI use for CUP in routine clinical practice, including patients with an ECOG PS of  ≥ 2, who were excluded from the pivotal clinical trial [9]. Although several real-world studies have previously reported on ICI use in CUP, they involved smaller cohorts [7, 10–14].

The incidence of TRAEs and Grade ≥ 3 TRAEs in this PMS (25.2% and 11.9%) was numerically lower than that reported in the NM-K2002 trial (any grade: 62.5%; Grade ≥ 3: 19.6%) (Fig. 2) [9, 15]. When compared with clinical trials, the incidence of TRAEs was similarly low in PMS studies of nivolumab for other carcinomas, including renal cell carcinoma, malignant melanoma, head and neck cancer, and NSCLC [5, 16–18]. Because safety monitoring in these PMS studies relies on routine clinical practice rather than protocol-driven assessments, the incidence of TRAEs may be underestimated compared with clinical trials.

TRAEs not observed in NM-K2002 but seen in this PMS included encephalitis and meningitis (any grade: 0.7%; Grade  ≥ 3: 0.7%), and type 1 diabetes mellitus (any grade: 0.7%; Grade ≥ 3: 0.7%). These events have been reported with nivolumab in other carcinomas at low frequencies (≤ 1% for both TRAEs of any grade and Grade ≥ 3 TRAEs) [18, 19], and, therefore, no new safety signals were identified. Subgroup analyses of elderly patients and those with pulmonary or thyroid disease also did not identify new safety concerns, consistent with real-world studies of nivolumab and other ICIs in similar higher-risk patient populations across multiple tumor types [19–22].

Regarding the effectiveness analysis, the overall ORR was consistent with that reported in NM-K2002 [9]. We conducted exploratory subgroup analyses for ORR, and a trend similar to that in NM-K2002 was observed in patients with lymph node-only involvement. In contrast, this PMS showed a numerically higher ORR in patients without prior systemic therapy than in those with prior systemic therapy, representing a difference from the clinical trial. This pattern has also been reported with nivolumab in head and neck cancer [23] and urothelial carcinoma [24], as well as with pembrolizumab in melanoma [25, 26], raising the hypothesis that nivolumab may be associated with greater immunotherapy sensitivity in CUP patients without prior systemic therapy compared with those who have received systemic therapy. Several biological mechanisms may account for this observation. CUP has been hypothesized to arise through immune-mediated regression of the primary tumor, while metastatic sites acquire immune-evasion mechanisms [4, 7]. CUP also frequently shows high programmed death-ligand 1 (PD-L1) expression at levels comparable to tumor types for which nivolumab is approved [8]. In addition, prior chemotherapy or radiotherapy may induce resistance, reducing ICI efficacy in pretreated patients [27]. These findings support our hypothesis that systemic treatment-naïve patients with CUP may derive more benefit from nivolumab. However, findings from the NM-K2002 study appear inconsistent with this hypothesis, potentially reflecting the variability due to the small sample size [9]. Larger, prospective studies are needed to test this hypothesis.

This PMS included exploratory subgroup analyses not conducted in the clinical trial, including analyses by histology, ECOG PS, and age. By histology, ORR was numerically higher in patients with squamous cell carcinoma and poorly differentiated malignant neoplasms than in those with adenocarcinoma. Previous studies have reported that squamous cell CUP exhibits higher tumor mutational burden (TMB) and PD-L1 expression levels than non-squamous CUP [4]. Furthermore, in NM-K2002, patients with CUP and high TMB or PD-L1 expression showed greater antitumor activity with nivolumab [9]. The reason for the higher response observed in poorly differentiated malignancies remains unclear, as these tumors represent a heterogeneous group with diverse biological features. Nevertheless, similar associations between high grade or poorly differentiated histology and greater benefit from ICIs have been reported in head and neck squamous cell carcinoma [28], neuroendocrine neoplasms [29], and sarcomatoid variant renal cell carcinoma [30]. In these tumor types, high TMB and/or elevated PD-L1 expression have been reported and are considered to contribute, at least in part, to enhanced responsiveness to ICIs.

Recent genomic studies in metastatic cancers and CUP have suggested that not only TMB but also copy number variation (CNV) may influence outcomes with ICI. Tumors with high TMB and low CNV levels tend to exhibit the most favorable responses to ICIs in pan-cancer analyses, and CNV loss has prognostic relevance in CUP [31, 32]. Although molecular data were not available in this study, such biomarker-driven classification may refine future patient selection and should be explored in future prospective studies.

This PMS also provided insights into the association between ECOG PS and ORR. Consistent with data from other malignancies [33–36] and studies of nivolumab plus ipilimumab in CUP [37], a trend toward higher ORR was observed in patients with better ECOG PS.

In this PMS, the ORR was numerically higher in patients aged < 75 years; however, previous reports have generally shown no clear association between ICI efficacy and age [38]. Therefore, the observed difference in ORR may be due to variations in patients’ immune profile [7].

Exploratory subgroup analyses of OS showed differences according to ECOG PS and the presence of lymph node-only involvement. A similar association for lymph node-only involvement has been reported in NM-K2002 [9], and ECOG PS has likewise been associated with OS in patients with CUP treated with nivolumab plus ipilimumab [37]. Both ECOG PS and lymph node-only involvement are recognized prognostic factors in clinical guidelines for CUP [1, 39], which may partly account for the observed differences in OS in this PMS.

This analysis has several limitations. The absence of control groups, short follow-up period, and lack of central review for safety and effectiveness evaluations require careful interpretation of results. As with other PMS studies, there was a risk of misclassification or under-reporting of TRAEs. In addition, subgroup analyses were exploratory, and potential imbalances in baseline characteristics among subgroups may have influenced the observed results. Tumor PD-L1 expression was not assessed.

## Conclusions

This is the first Japanese PMS of nivolumab monotherapy for CUP. Nivolumab was tolerated, with no new safety signals, and indicated effectiveness consistent with clinical trial data. These findings support nivolumab as a therapeutic option for patients with CUP in real-world practice.

## Supplementary Information

Below is the link to the electronic supplementary material.

Supplementary file 1 (PDF 406 KB)

## Data Availability

The datasets generated and/or analyzed during the current study are not publicly available because patient consent for individual data disclosure was not obtained, but are available from the corresponding author on reasonable request.
