# Supplementary material for: Real-world safety and effectiveness of nivolumab in patients with cancer of unknown primary: results from a nationwide post-marketing surveillance in Japan
Source: Int J Clin Oncol. 2026 Aug 26;31(10):2363–73. doi: 10.1007/s10147-026-03147-3 (PMC13615158; doi:10.1007/s10147-026-03147-3)
Supplement: Supplementary file 1 [file 10147_2026_3147_MOESM1_ESM.pdf]

Supplementary materials

Real-world safety and effectiveness of nivolumab in patients with cancer of unknown primary: results from a nationwide post-marketing surveillance in Japan

Akira Kawasaki, Akito Kakuuchi, Ayumi Akamatsu, Misato Nakanishi, Chikara Honda

Corresponding author: Ayumi Akamatsu

Email: a.akamatsu@ono-pharma.com

**Content**	**Page**
**Supplementary Table S1** Treatment exposure and dosing of nivolumab (*n*=151)	2
**Supplementary Table S2** Incidence of TRAEs according to patient subgroups (*n*=151)	3
**Supplementary Table S3** Clinical outcomes and treatments of selected TRAEs (*n*=151)	6
**Supplementary Table S4** DCR according to patient subgroups (*n*=144)	7
**Supplementary Fig. S1** Kaplan–Meier plots of OS according to ECOG PS (*n*=144)	8
**Supplementary Fig. S2** Kaplan–Meier plots of OS according to age at the start of nivolumab (*n*=144)	9
**Supplementary Fig. S3** Kaplan–Meier plots of OS in patients with/without prior systemic therapy before nivolumab (*n*=144)	10
**Supplementary Fig. S4** Kaplan–Meier plots of OS according to the tumor histologic type (*n*=144)	11
**Supplementary Fig. S5** Kaplan–Meier plots of OS according to the lesion site at the start of nivolumab (*n*=144)	12

Supplementary Table S1 Treatment exposure and dosing of nivolumab (*n*=151)

**Parameter**		**Patients (%)**
Frequency of administration	1–4 times	67 (44.4)
	5–8 times	38 (25.2)
	9–12 times	30 (19.9)
	≥13 times	16 (10.6)
	Unknown	0
	Mean (SD)	6.2 (4.0)
	Median (range)	5.0 (1–14)
Duration of treatment	1–60 days	66 (43.7)
	61–120 days	29 (19.2)
	121–180 days	36 (23.8)
	≥181 days	20 (13.2)
	Unknown	0
	Mean (SD)	89.7 (66.1)
	Median (range)	73.0 (1–183)
Total dose of nivolumab	<480 mg	29 (19.2)
	>480–<960 mg	30 (19.9)
	>960–<1920 mg	33 (21.9)
	>1920–<2880 mg	31 (20.5)
	>2880 mg	28 (18.5)
	Unknown	0
	Mean (SD)	1697.5 (1063.3)
	Median (range)	1440.0 (240–3600)
Dose per administration	240 mg	102 (67.5)
	Switch from 240 mg to 480 mg	30 (19.9)
	480 mg	17 (11.3)
	Other	2 (1.3)
Discontinued nivolumab at 6 months	No	40 (26.5)
	Yes	111 (73.5)
Reasons for discontinuation[a]	Disease progression (including death)	64 (57.7)
	Lack of expected efficacy	29 (26.1)
	Adverse events	22 (19.8)
	Transfer to another facility	7 (6.3)
	Other	6 (5.4)

Values are *n* (%), unless specified otherwise

[a]Multiple reasons were possible

SD standard deviation

Supplementary Table S2 Incidence of TRAEs according to patient subgroups (*n*=151)

Patient characteristic		Patients	TRAEs	Grade ≥3 TRAEs[a]
All		151 (100)	38 (25.2)	18 (11.9)
Sex	Male	86 (57.0)	19 (22.1)	9 (10.5)
	Female	65 (43.0)	19 (29.2)	9 (13.8)
ECOG PS (0–4)	0	42 (27.8)	12 (28.6)	7 (16.7)
	1	78 (51.7)	18 (23.1)	8 (10.3)
	2	26 (17.2)	7 (26.9)	3 (11.5)
	3	4 (2.6)	1 (25.0)	0
	4	1 (0.7)	0	0
ECOG PS (0–1, 2–4)	0–1	120 (79.5)	30 (25.0)	15 (12.5)
	2–4	31 (20.5)	8 (25.8)	3 (9.7)
Age (15–<65, 65–<75, ≥75)	15–<65 years	50 (33.1)	6 (12.0)	2 (4.0)
	65–<75 years	47 (31.1)	14 (29.8)	6 (12.8)
	≥75 years	54 (35.8)	18 (33.3)	10 (18.5)
Age (<75, ≥75)	<75 years	97 (64.2)	20 (20.6)	8 (8.2)
	≥75 years	54 (35.8)	18 (33.3)	10 (18.5)
Inpatient/outpatient	Inpatient	49 (32.5)	9 (18.4)	3 (6.1)
	Outpatient	102 (67.5)	29 (28.4)	15 (14.7)
BMI	<18.5 kg/m^2	23 (15.2)	6 (26.1)	2 (8.7)
	18.5–<25 kg/m^2	97 (64.2)	26 (26.8)	13 (13.4)
	≥25 kg/m^2	26 (17.2)	5 (19.2)	3 (11.5)
	Unknown	5 (3.3)	1 (20.0)	0
Medical history	No	69 (45.7)	15 (21.7)	7 (10.1)
	Yes	82 (54.3)	23 (28.0)	11 (13.4)
Medical history: lung	No	143 (94.7)	33 (23.1)	14 (9.8)
	Yes	7 (4.6)	5 (71.4)	4 (57.1)
	Unknown	1 (0.7)	0	0
Medical history: liver	No	140 (92.7)	36 (25.7)	17 (12.1)
	Yes	10 (6.6)	2 (20.0)	1 (10.0)

	Unknown	1 (0.7)	0	0
Medical history: kidney	No	140 (92.7)	36 (25.7)	17 (12.1)
	Yes	10 (6.6)	2 (20.0)	1 (10.0)
	Unknown	1 (0.7)	0	0
Medical history: thyroid	No	144 (95.4)	34 (23.6)	17 (11.8)
	Yes	6 (4.0)	4 (66.7)	1 (16.7)
	Unknown	1 (0.7)	0	0
Medical history: autoimmune	No	137 (90.7)	34 (24.8)	17 (12.4)
	Yes	13 (8.6)	4 (30.8)	1 (7.7)
	Unknown	1 (0.7)	0	0
Medical history: other diseases	No	83 (55.0)	19 (22.9)	9 (10.8)
	Yes	68 (45.0)	19 (27.9)	9 (13.2)
Tumor histologic type	Well/moderately differentiated adenocarcinoma	39 (25.8)	10 (25.6)	7 (17.9)
	Poorly differentiated adenocarcinoma/undifferentiated carcinoma	47 (31.1)	5 (10.6)	1 (2.1)
	Squamous cell carcinoma	23 (15.2)	9 (39.1)	5 (21.7)
	Neuroendocrine tumor	2 (1.3)	1 (50.0)	0
	Poorly differentiated malignant neoplasm	18 (11.9)	6 (33.3)	3 (16.7)
	Other tumors	22 (14.6)	7 (31.8)	2 (9.1)
Lesion site at the start of nivolumab by organ[b]	Lymph-node	85 (56.3)	22 (25.9)	9 (10.6)
	Mediastinum	7 (4.6)	2 (28.6)	1 (14.3)
	Lung	16 (10.6)	4 (25.0)	1 (6.3)
	Pleura	13 (8.6)	5 (38.5)	3 (23.1)
	Peritoneum	53 (35.1)	10 (18.9)	6 (11.3)
	Retroperitoneum	4 (2.6)	2 (50.0)	1 (25.0)
	Liver	23 (15.2)	4 (17.4)	2 (8.7)
	Bone	32 (21.2)	5 (15.6)	2 (6.3)
	Brain	2 (1.3)	0	0
	Others	24 (15.9)	7 (29.2)	1 (4.2)

Prior surgical treatment for CUP	No	134 (88.7)	38 (28.4)	18 (13.4)
	Yes	17 (11.3)	0	0
Prior radiation therapy for CUP	No	118 (78.1)	28 (23.7)	13 (11.0)
	Yes	33 (21.9)	10 (30.3)	5 (15.2)
Prior systemic therapy before nivolumab	No	73 (48.3)	14 (19.2)	8 (11.0)
	Yes	78 (51.7)	24 (30.8)	10 (12.8)
Number of systemic therapy regimens before nivolumab	0	73 (48.3)	14 (19.2)	8 (11.0)
	1	56 (37.1)	19 (33.9)	9 (16.1)
	2	12 (7.9)	2 (16.7)	1 (8.3)
	≥3	10 (6.6)	3 (30.0)	0
Prior immunotherapy for CUP	No	151 (100.0)	38 (25.2)	18 (11.9)
	Yes	0	0	0
Combination therapy (surgery)	No	149 (98.7)	37 (24.8)	18 (12.1)
	Yes	2 (1.3)	1 (50.0)	0
Combination therapy (radiation therapy)	No	140 (92.7)	35 (25.0)	15 (10.7)
	Yes	11 (7.3)	3 (27.3)	3 (27.3)
Concomitant drug (antineoplastic drug)	No	147 (97.4)	38 (25.9)	18 (12.2)
	Yes	4 (2.6)	0	0
Concomitant drug (vaccine)	No	142 (94.0)	34 (23.9)	17 (12.0)
	Yes	9 (6.0)	4 (44.4)	1 (11.1)
Lesion site at the start of nivolumab	Lymph node–only involvement[c]	41 (27.2)	14 (34.1)	6 (14.6)
	Lesions at other sites, with or without lymph node involvement	110 (72.8)	24 (21.8)	12 (10.9)

Values are *n* (%), unless otherwise stated

[a]Using the total of Grades 3, 4, and 5 as the numerator

[b]If multiple lesion sites were recorded at the start of nivolumab, each site was counted

[c]Patients with no lesions at sites other than lymph node(s)

BMI body mass index, *CUP* cancer of unknown primary, *ECOG PS* Eastern Cooperative Oncology Group performance status, *TRAE* treatment-related adverse event

Supplementary Table S3 Clinical outcomes and treatments of selected TRAEs (*n*=151)

	Recovered or recovering	Recovered with sequalae	Non-recovery	Death	Unknown	Total
ILD	7 (100.0)	0	0	0	0	7
No treatment	2 (100.0)	0	0	0	0	2
Treated with a corticosteroid	5 (100.0)	0	0	0	0	5
Hepatic failure, hepatic dysfunction, hepatitis, and cholangitis sclerosing	6 (75.0)	0	1 (12.5)	0	1 (12.5)	8
Untreated	4 (80.0)	0	0	0	1 (20.0)	5
Treated	2 (66.7)	0	1 (33.3)	0	0	3
Treated with a corticosteroid	2 (100.0)	0	0	0	0	2
Treated with a ursodeoxycholic acid	0	0	1 (100.0)	0	0	1
Endocrine disorders	2 (28.6)	1 (14.3)	4 (57.1)	0	0	7
Untreated	0	1 (50.0)	1 (50.0)	0	0	2
Treated	2 (40.0)	0	3 (60.0)	0	0	5
Corticosteroid	2 (66.7)	0	1 (33.3)	0	0	3
Hormone replacement therapy, etc.	0	0	2 (100.0)	0	0	2

Values are *n* (%), with percentages calculated using the number of patients in each category as the denominator

ILD interstitial lung disease, *TRAE* treatment-related adverse event

Supplementary Table S4 DCR according to patient subgroups (*n*=144)

**Patient characteristic**		**Patients, *n* (%)**	**Patients with CR, PR, or SD, *n*[a]**	**DCR (95% CI)**
All		144 (100)	67	46.5 (38.2–55.0)
ECOG PS	0–1	114 (79.2)	62	54.4 (44.8–63.7)
	2	25 (17.4)	5	20.0 (6.8–40.7)
	3–4	5 (3.5)	0	0 (0.0–52.2)
Age	<75 years	93 (64.6)	44	47.3 (36.9–57.9)
	≥75 years	51 (35.4)	23	45.1 (31.1–59.7)
Prior systemic therapy before nivolumab	No	67 (46.5)	32	47.8 (35.4–60.3)
	Yes	77 (53.5)	35	45.5 (34.1–57.2)
Tumor histologic type	Well/moderately differentiated adenocarcinoma	37 (25.7)	13	35.1 (20.2–52.5)
	Poorly differentiated adenocarcinoma/ undifferentiated carcinoma	45 (31.3)	17	37.8 (23.8–53.5)
	Squamous cell carcinoma	22 (15.3)	15	68.2 (45.1–86.1)
	Neuroendocrine tumor	2 (1.4)	2	100.0 (15.8–100.0)
	Poorly differentiated malignant neoplasm	17 (11.8)	11	64.7 (38.3–85.8)
	Other tumors	21 (14.6)	9	42.9 (21.8–66.0)
Lesion site at the start of nivolumab	Lymph node-only involvement[a]	41 (28.5)	29	70.7 (54.5–83.9)
	Lesions at other sites, with or without lymph node involvement	103 (71.5)	38	36.9 (27.6–47.0)

[a]Patients with no lesions at sites other than lymph node(s)

CI confidence interval, *CR* complete response, *DCR* disease control rate, *ECOG PS* Eastern Cooperative Oncology Group performance status, *PR* partial response, *SD* stable disease

Supplementary Fig. S1 Kaplan–Meier plots of OS according to ECOG PS (*n*=144)

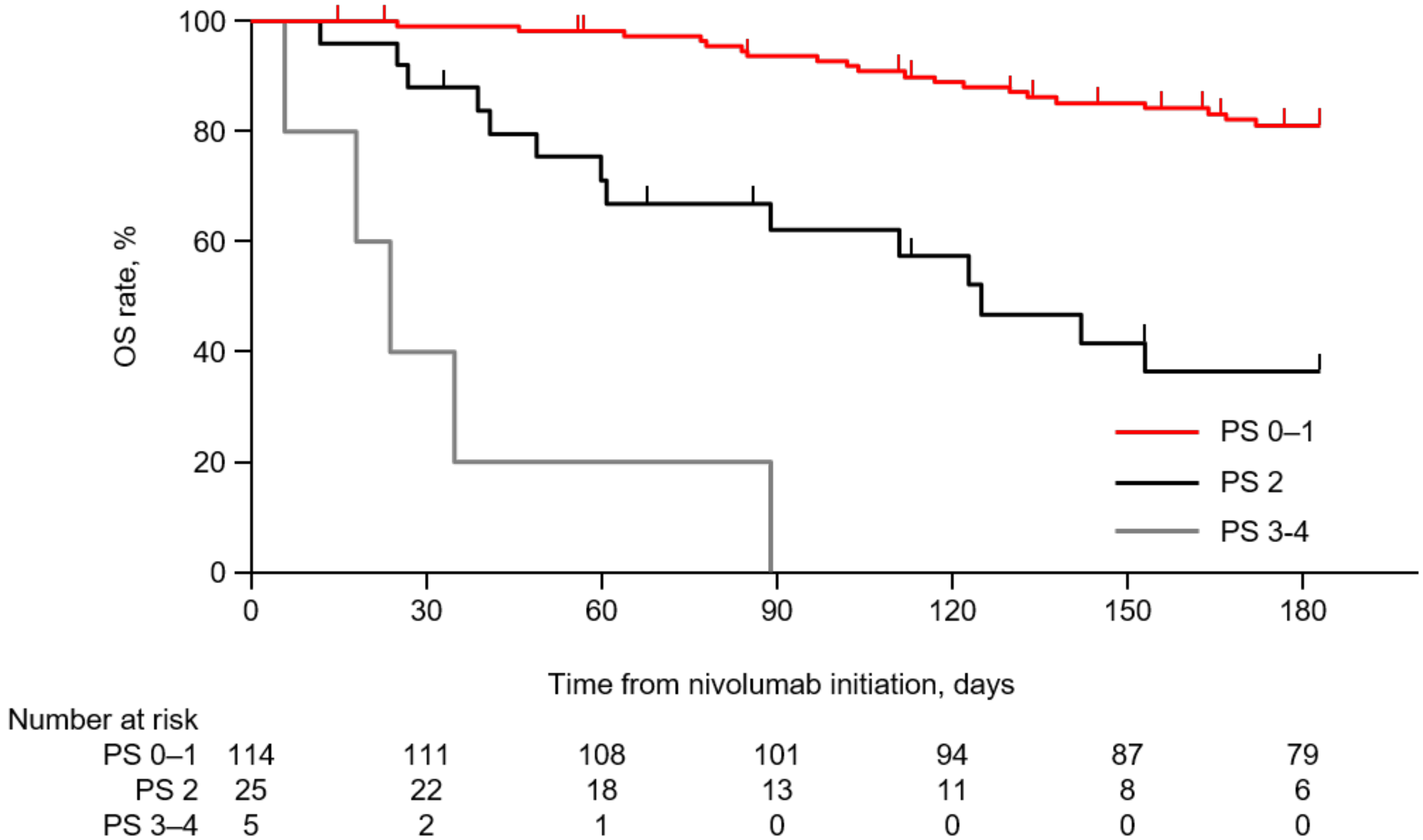

	6-month OS rate (95% CI), %	Median OS (95% CI), day
PS 0–1	81.2 (72.3–87.4)	NR (NR–NR)
PS 2	36.6 (17.0–56.5)	125.0 (60.0–NR)
PS 3–4	NR (NR–NR)	24.0 (6.0–NR)

CI confidence interval, *ECOG PS* Eastern Cooperative Oncology Group performance status, *NR* not reached, *OS* overall survival

Supplementary Fig. S2 Kaplan–Meier plots of OS according to age at the start of nivolumab (*n*=144)

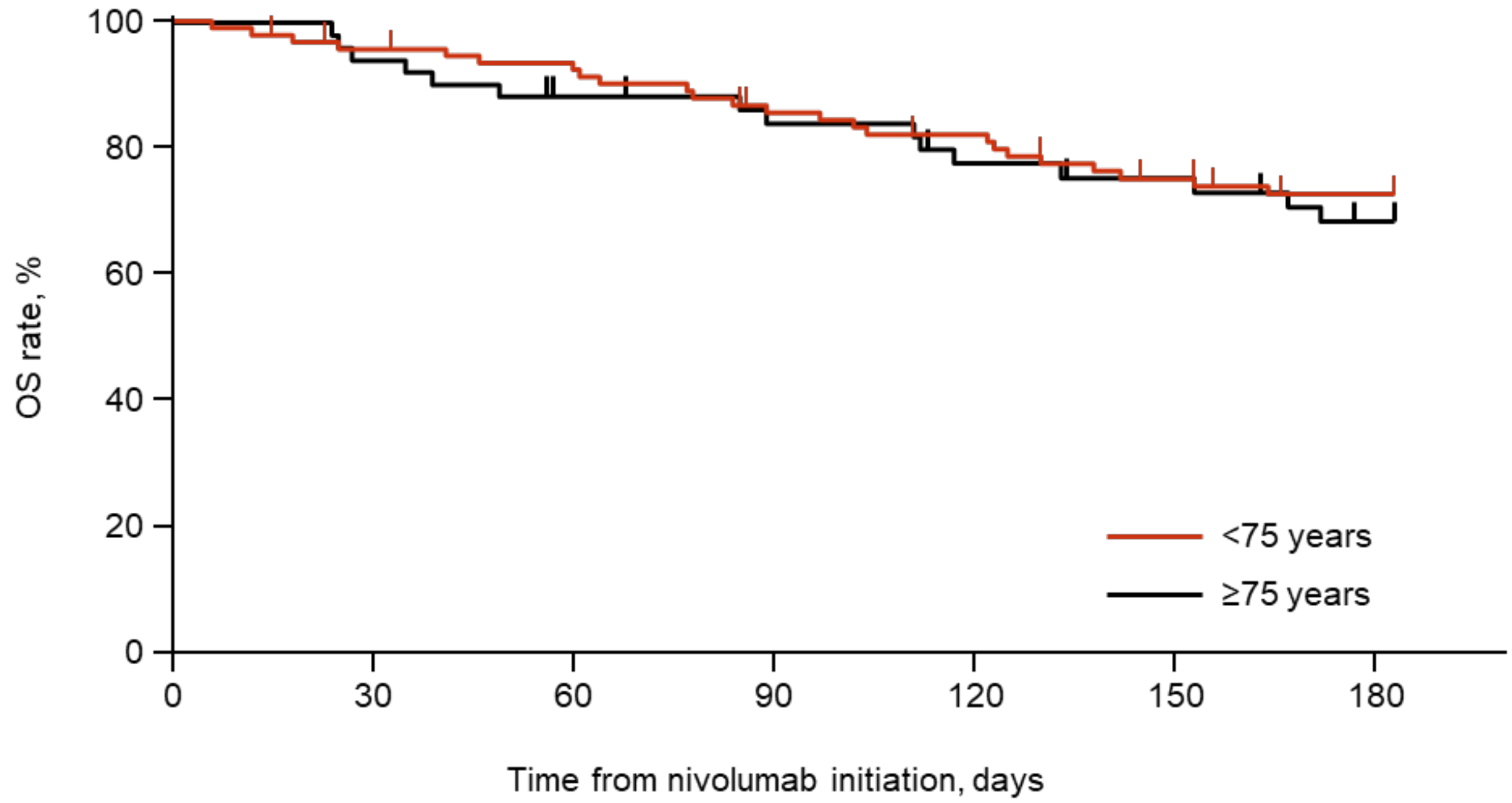

Number at risk							
<75 years	93	87	84	74	70	62	57
≥75 years	51	48	43	40	35	33	28

	**6-month OS rate (95% CI), %**	**Median OS (95% CI), day**
<75 years	72.6 (61.9–80.7)	NR (NR–NR)
≥75 years	68.4 (53.0–79.7)	NR (NR–NR)

CI confidence interval, *NR* not reached, *OS* overall survival

Supplementary Fig. S3 Kaplan–Meier plots of OS in patients with/without prior systemic therapy before nivolumab (n=144)

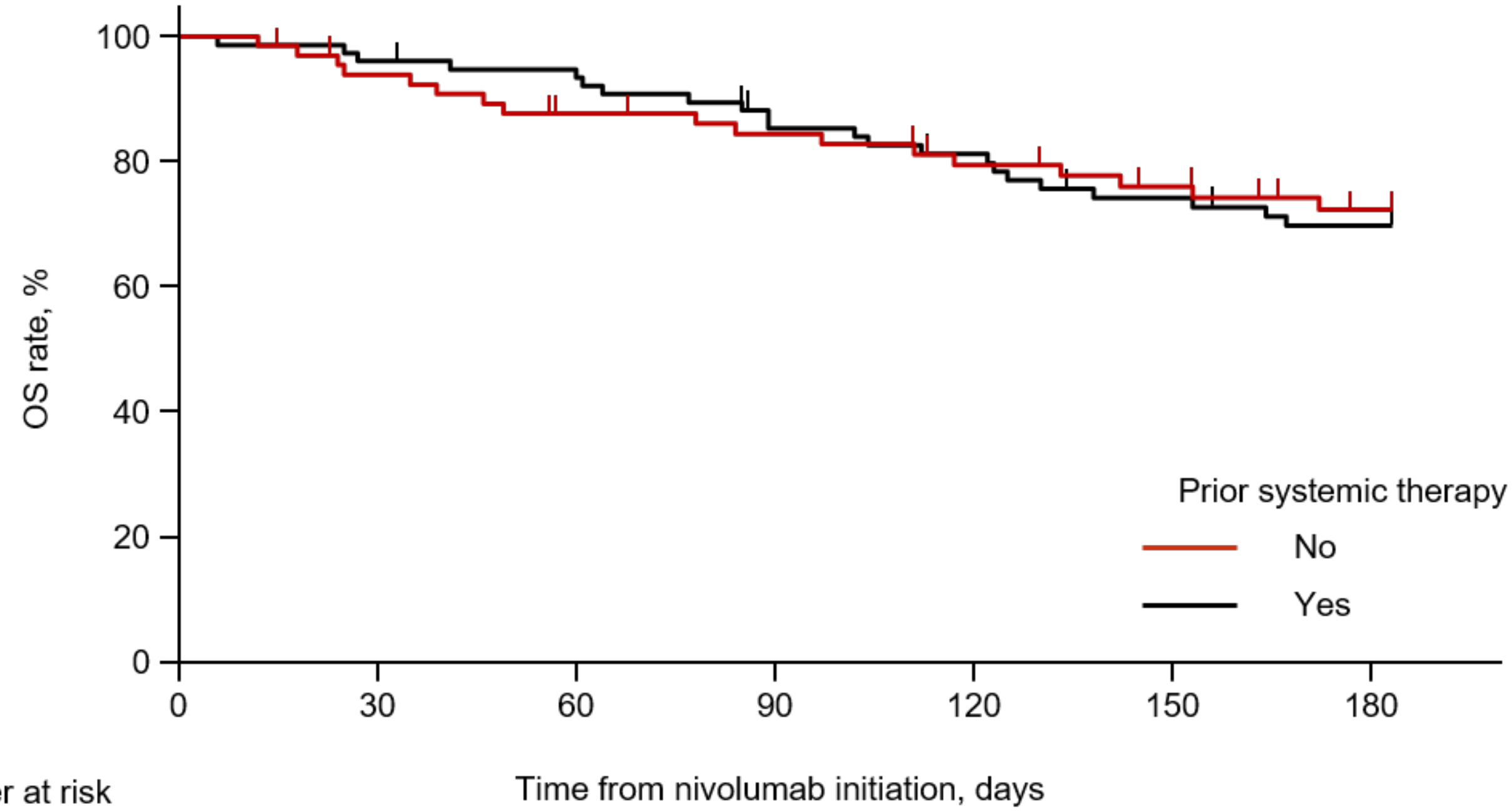

Number at risk<br>Prior systemic therapy	0	30	60	90	120	150	180
No	67	61	55	52	47	43	37
Yes	77	74	72	62	58	52	48

**Prior systemic therapy**	**6-month OS rate (95% CI), %**	**Median OS (95% CI), day**
No	72.4 (59.3–81.9)	NR (NR–NR)
Yes	69.9 (58.0–79.1)	NR (NR–NR)

CI confidence interval, *NR* not reached, *OS* overall survival

Supplementary Fig. S4 Kaplan–Meier plots of OS according to the tumor histologic type (*n*=144)

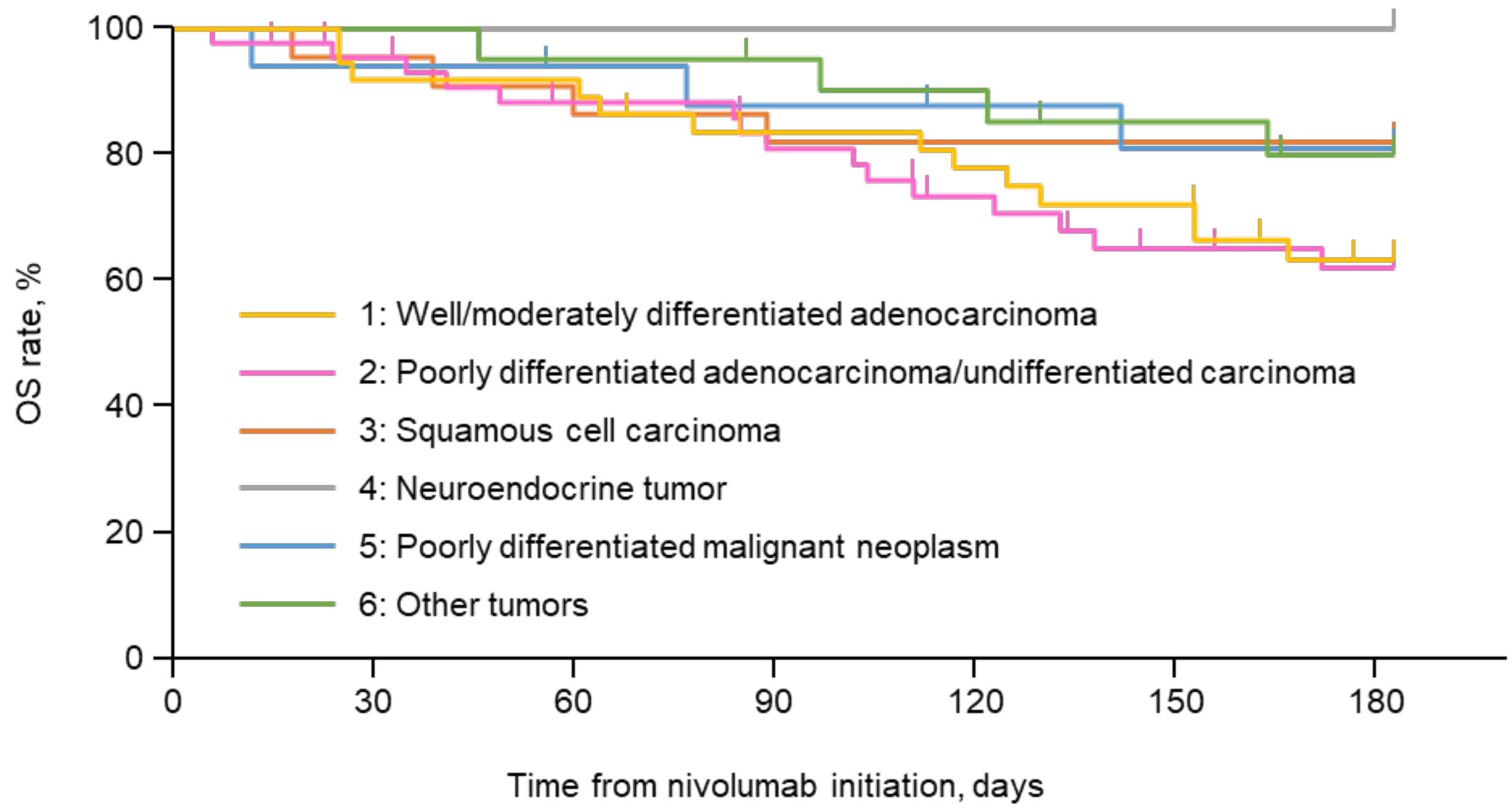

Number at risk

	0	30	60	90	120	150	180
1	37	34	34	29	27	25	19
2	45	41	36	32	27	22	20
3	22	21	20	18	18	18	18
4	2	2	2	2	2	2	2
5	17	16	15	14	13	12	12
6	21	21	20	19	18	16	14

	**6-month OS rate (95% CI), %**	**Median OS (95% CI), day**
1: Well/moderately differentiated adenocarcinoma	63.2 (45.1–76.8)	NR (153.0–NR)
2: Poorly differentiated adenocarcinoma/ undifferentiated carcinoma	61.9 (44.7–75.2)	NR (138.0–NR)
3: Squamous cell carcinoma	81.8 (58.5–92.8)	NR (NR–NR)
4: Neuroendocrine tumor	100.0 (100.0–100.0)	NR (NR–NR)
5: Poorly differentiated malignant neoplasm	81.1 (51.9–93.5)	NR (NR–NR)
6: Other tumors	79.9 (54.8–92.0)	NR (NR–NR)

CI confidence interval, *NR* not reached, *OS* overall survival

Supplementary Fig. S5 Kaplan–Meier plots of OS according to the lesion site at the start of nivolumab (*n*=144)

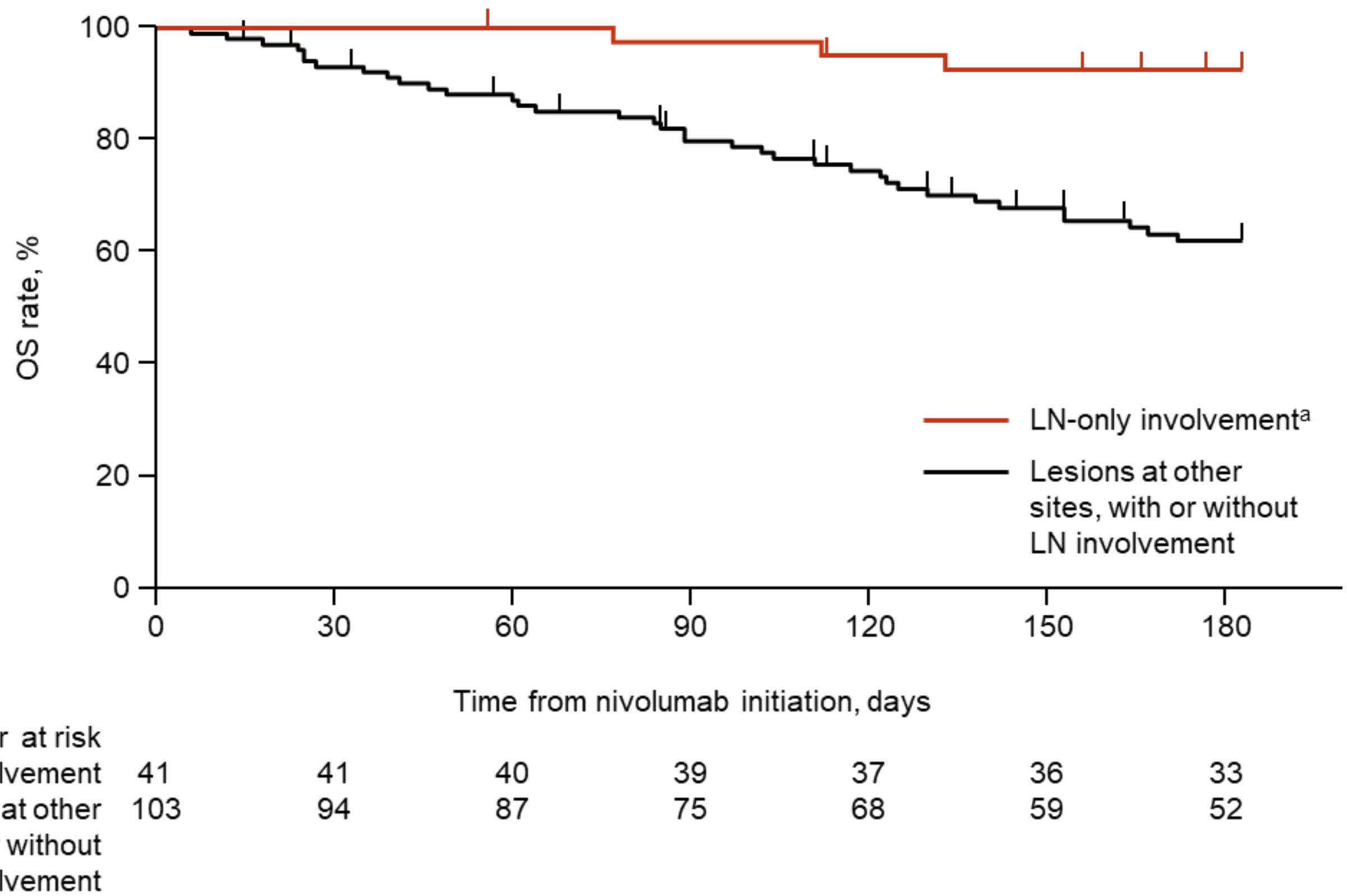

	6-month OS rate (95% CI), %	Median OS (95% CI), day
LN-only involvement[a]	92.4 (78.3–97.5)	NR (NR–NR)
Lesions at other sites, with or without LN involvement	62.0 (51.3–71.0)	NR (NR–NR)

[a] Patients with no lesions at sites other than lymph node(s)

CI confidence interval, *LN* lymph node, *NR* not reached, *OS* overall survival
